# Decreased consumption of natural rewards in rhesus monkeys with prolonged methamphetamine abstinence

**DOI:** 10.3389/fpsyt.2024.1446353

**Published:** 2024-09-06

**Authors:** Jiahui Zhou, Hang Su, Chengjie Tang, Xiaotian Wu, Zijing Wang, Wenlei Zhang, Rongwei Zhai, Haifeng Jiang

**Affiliations:** ^1^ Shanghai Mental Health Center, Shanghai Jiao Tong University School of Medicine, Shanghai, China; ^2^ Department of Disease Biology, Lingang Laboratory, Shanghai, China

**Keywords:** methamphetamine, abstinence, consumption, improved sucrose preference test, sucrose

## Abstract

**Rationale:**

Relapse to drug use is a major clinical challenge in the treatment of addictive disorders, including psychostimulant use and may be exacerbated by reduced sensitivity to natural, non-drug reward. Given the relatively limited set of outcomes, and short withdrawal time in rodent studies, we conducted a more detailed assessment of the response to natural rewards in methamphetamine (METH) naive versus exposed monkeys during long-term abstinence.

**Methods:**

This study introduced an improved sucrose preference test (iSPT) to assess natural reward seeking and consumption in monkeys with long-term abstinence after methamphetamine (METH) use. The test was administered to sixteen naive monkeys and five METH exposed monkeys that had been abstinent for at least 3 months.

**Results:**

METH exposed monkeys showed a lower sucrose preference score in both the iSPT (z = -2.10, p = 0.036) and the sucrose preference test (z = -2.61, p = 0.009). The sucrose preference score was significantly correlated with the latency of the establishment of stable sucrose-preference (r = -0.76, df = 46, p < 0.001) but not with the other variables. Furthermore, water-sucrose switch latency and switch times were significantly negatively correlated (r = -0.50, df = 20, p = 0.02).

**Conclusion:**

These results show reductions in natural reward consumption during long-term methamphetamine abstinence.

## 1 Introduction

Substance use disorders (SUD) cause high psychosocial costs and represent a substantial burden to public health (1). A dataset from the National Inpatient Sample (NIS) of the United States (US) showed that the prevalence of drug abuse and psychiatric disorders among hospitalized patients increased in 2017 compared to 2007 (2). In addition, the social costs related to the use of methamphetamine (METH) were extremely high and estimated at Australian Dollar (AUD) 5,023.8 million in Australia in 2013/14 (3). Following METH detoxification, relapse rates within one year were high, with only 23% of subjects remaining abstinent one year after terminated METH use (4). Impaired seeking of alternative rewards in individuals with chronic drug abuse may result in relapse by hindering the pursuit of adaptive alternatives to drug use, especially after withdrawal (5–7). In accordance with this hypothesis, a study revealed that smokers who exhibited diminished levels of brain reactivity towards pleasant stimuli, compared to cigarette-related cues, had a higher probability of relapsing within six months following their attempt to quit (8). In animal studies, rats treated with high doses of METH exhibited significant decreases in both the volume of liquid consumed and their preference for the sucrose-containing bottle following 25 days withdrawal (9). Mice also displayed a decline in sucrose preference scores after one week withdrawal from chronic METH exposure (10). Regarding amphetamine withdrawal, Vacca et al. (2007) observed reduced dopamine release elicited by cues during the preparatory phase of sucrose consumption, suggesting that a blunted mesocorticolimbic dopamine function may impair motivation for the non-drug reward sucrose (11). However, one other study showed that the exposure and withdrawal didn’t lead to lower sucrose preference but discounting of reward value by effort (12). Given the limited findings and duration of abstinence from psychostimulants in rodent studies, we aimed to conduct a more detailed assessment of the response to natural rewards in non-human primates who had been withdrawn from METH use for an extended period of time.

In this study, we assessed naive monkeys and monkeys with long-term abstinence following METH exposure. To measure natural (non-drug) reward seeking and consumption, we modified an established sucrose preference test (13), and named it the improved sucrose preference test (iSPT). We applied a traditional sucrose preference test (SPT) as well as the iSPT that gave animals more time to get familiar with testing conditions. We assessed a sucrose preference score, total liquid consumption, the latency to the first drink, water-sucrose switch latency, switch times and the latency of the establishment of stable sucrose-preference. METH exposed monkeys were assessed after at least three months of abstinence, to exclude any impact of METH withdrawal; in fact, most withdrawal symptoms are largely resolved within approximately three weeks of abstinence (14, 15).

We hypothesized that METH exposed monkeys displayed a lower sucrose preference score, which reflects reward consumption (16). We further investigated whether reward-seeking behavior was impaired, which might be reflected in a prolonged latency to switch from water to sucrose and a longer duration required to establish a stable sucrose preference. Finally, we tested the number of switches between water and sucrose in the METH abstinent versus naive monkeys.

## 2 Materials and methods

### 2.1 Subjects

A total of 21 male rhesus monkeys (*Macaca mulatta*) were divided into two distinct groups. The METH group (n = 5) consisted of monkeys with a history of chronic METH self-administration (69 ± 44 mg/kg for 5.6 ± 3.4 months). The naive group (n = 16) consisted of experimentally naïve monkeys that had not been previously exposed to METH or saline.

All monkeys were separately housed within steel cages, which ensured visual, auditory and olfactory contact with conspecifics for the duration of the study. The cage utilized in our study measured 100 cm x 90 cm x 90 cm. All the animals were arranged on a 12-h light/dark cycle (at 07:00) with fresh fruits at noon, approximately 500 ml of water, and 150 g of monkey chow daily for more than one year before testing. Toys were provided in their cages, and videos were played one to two times weekly in the room to offer additional environmental stimulation. Experimental procedures were reviewed and supported by the Animal Care Committee of the Institute of Neuroscience and Center for Excellence in Brain Science and Intelligence Technology, Chinese Academy of Sciences. Animal care followed the US National Institutes of Health Guide for the Care and Use of Laboratory Animals. The ethics approval number is (#ION-2017006).

### 2.2 Apparatus and drugs

All behavioral tests were executed in the respective home cages. All bottles and boxes were provided by Suzhou Houhuang Animal Experimentation Equipment Co., Ltd. METH (> 99% purity) was offered by the Center for Excellence in Brain Science and Intelligence Technology, and 0.9% saline solution was used to dissolve the drug.

### 2.3 Experimental protocol

#### 2.3.1 METH self-administration

METH application was described in detail in a previous publication (17). In short, before withdrawal and long-term abstinence, the monkeys in the METH group were provided with two levers associated with juice and methamphetamine rewards, respectively. A white light indicated the onset of a trial. Two levers were available, pressing them resulted in delivery of 2 ml juice or an intravenous infusion of 0.032 mg/kg METH. Once the lever press was enough, the white light was terminated and, depending on whether METH (red light) or juice (green light) was delivered, a different light stimulus above the respective lever and a tone stimulus (10 s) corresponding to the lever were presented to the monkeys. Monkeys were permitted to self-administer METH for a duration of 2 hours during each session, with a total of 5 sessions per week. The average METH intake of the 5 monkeys was 69 ± 44 mg/kg.

#### 2.3.2 Sucrose preference test

**Figure 1:**
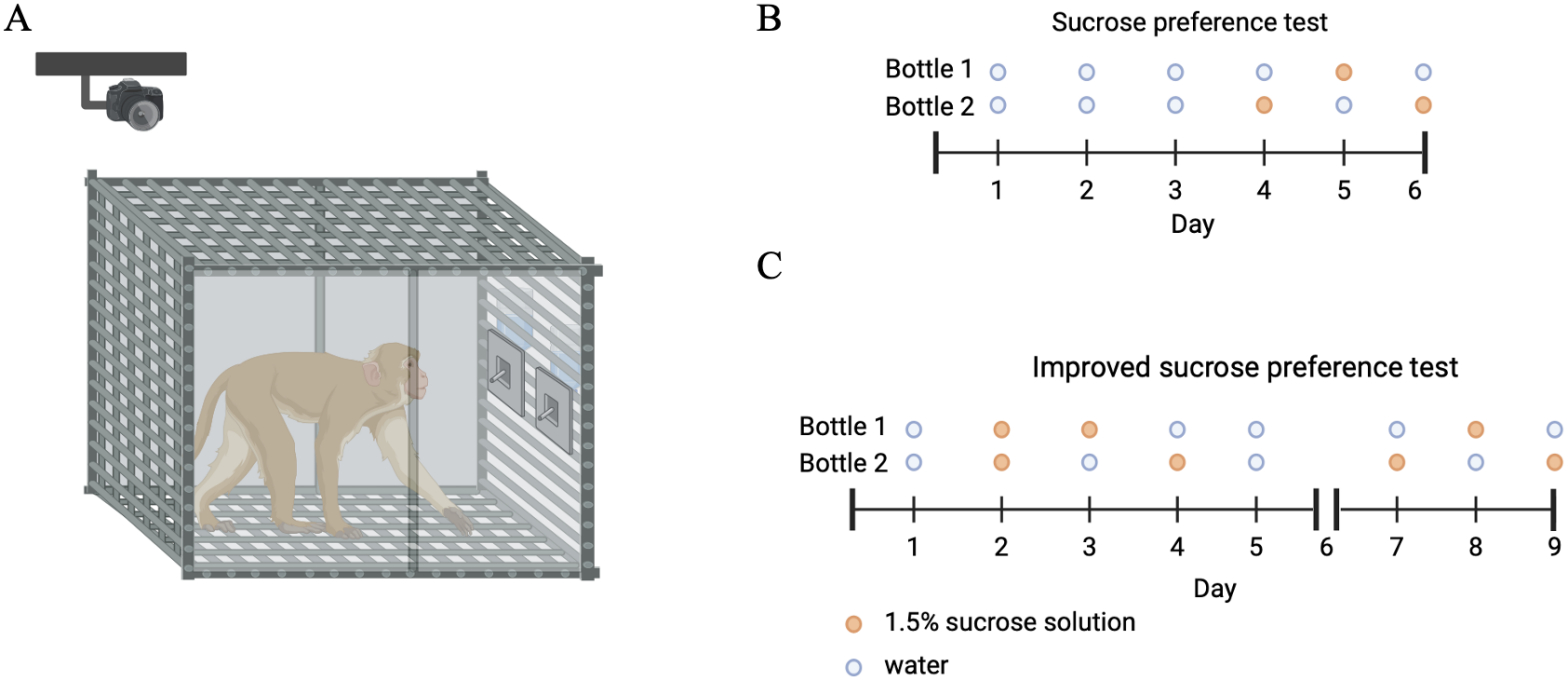
Overview of the experimental protocol. **(A)** The monkey was presented with two identical bottles with different solutions. **(B)** Bottles were filled with a 1.5% sucrose solution (orange) and pure water (blue) according to the procedures of the sucrose preference test. **(C)** The procedures of the improved sucrose preference test. The picture was created by Biorender.

A traditional sucrose preference test was applied to validate our method (13, 18). First, monkeys were trained to drink from two bottles which contained pure water on three consecutive days. After that, the monkeys underwent a water-deprivation phase for 23 hours. Then, on the initial day of the testing phase, the monkeys could drink pure water or 1.5% sucrose solution from two identically looking bottles for 1 hour at 13:00 PM. During the session, we replenished the two bottles at 30 minutes, and measured the total consumption of water and sucrose solution at the end of the session. On each of the next two days of the testing phase, again at 13:00 PM, the locations of the two bottles with the different liquids were interchanged (see **Figure 1**). For every session, sucrose preference score was calculated as the ratio of the sucrose consumption to the total consumption. Total liquid consumption was calculated as the ratio of the total volume consumed to the subject’s body weight.

#### 2.3.3 Improved sucrose preference test

We revised the traditional sucrose preference test (see **Figure 1**). This test includes three stages: 1) the habituation stage; 2) a no-water period; and 3) a testing period. During these testing phases, two bottles were placed in nontransparent boxes, with the spouts around 35 cm from the cage floor and about 10-20 cm adjacent to the other. In addition, a 1.5% (wt/vol) sucrose solution was prepared in advance and used within two days.

On the initial day of the five-day habituation stage, monkeys had access to two identical bottles in terms of both size and color. Each bottle was filled with 500 ml of pure water. Subsequently, on the second day at 14:00 PM, the remaining fluid in each bottle was carefully measured using a gauged cylinder before being replaced in both bottles with a solution of 1.5% sucrose, which ensured equal capacity. On the third day, one of the bottles contained pure water while the other one contained the sucrose solution, following the same procedures as the preceding day. On the fourth day, manipulation was introduced whereby the locations of the two bottles with water or sucrose solution were interchanged. The presentation with two bottles containing sucrose solutions and the switches of locations were done to make the monkeys were aware of the availability of the sweet reward and the contingencies associated with the sucrose solution. On the fifth day, both bottles were once again filled with 500 ml of pure water. This was aimed at decreasing any potential impacts of prior sucrose intake.

During the no-water period directly before the testing period, monkeys underwent a water-deprivation phase that commenced at 14:00 PM and lasted until the same time the following day (13:00 PM).

Finally, during the testing stage encompassing three sessions, each session adhered to the same procedural protocols as detailed in testing phase of sucrose preference test.

For every session, a sucrose preference score was calculated as the ratio of the sucrose consumption to the total fluid consumption. We also assessed time spent on the bottle containing sucrose during the first 5 minutes, 10 minutes and 1 hour per session. This drinking was assessed by video surveillance and defined as the monkey’s mouth touching the spout, e.g. for sucking or licking. Sucrose-related preference was determined as the ratio of the drinking time spent on the sucrose-related bottle to the total drinking time and correlated with total sucrose intake during the same session.

### 2.4 Behavioral observations

Fixed cameras were installed on cages. Behavior of monkeys was recorded via video documentation during the hour subsequent to the presentation of the liquids. Drinking was defined as the monkey’s mouth touching the spout, such as sucking or licking. Subsequently, the recorded behaviors were analyzed by a worker who remained unaware of the precise location of the sucrose solution, which aimed to minimize any potential bias in the assessment process.

### 2.5 Water-sucrose switch latency

Since bottles were identical, either water or sucrose would be randomly selected by monkeys to consume first. During the sessions in which the monkey initially chose to consume water, the water-sucrose switch latency was measured and operationally defined as the time interval between the start of the initial water consumption and the subsequent start of drinking the sucrose solution.

### 2.6 Latency of the establishment of stable sucrose-preference

Each one-hour session was divided into 60 units (each unit lasting for 1 minute). Sucrose preference in this one unit was recorded if the monkey spent at least two-thirds of the total drinking on the sucrose-related bottle and calculated as the ratio of the drinking time spent on the sucrose-related bottle to the total drinking time (19). The time point of the establishment of a “stable” sucrose-preference was defined as the first minute within a consecutive five-minute interval of drinking in which there was less than 10% variation of sucrose preference for the sucrose-related bottle in five consecutive one-minute drinking time units (20–22). In cases a monkey did not exhibit a stable sucrose preference throughout the whole testing session, latency time was assigned a value of 60 min.

### 2.7 Switch times

Switch times were calculated as the cumulative number of place switches between the bottle with water and the one with sucrose solution throughout the entire session.

### 2.8 Latency to the first drink

Latency to the first drink was defined as the time interval between the presentation of two bottles and the first drinking behavior.

### 2.9 Statistical analysis

Statistical analysis was performed by use of International Business Machines Corporation (IBM) Statistical Package for the Social Sciences (SPSS) Statistics for Mac, Version 27.0 (IBM Corp, Armonk, New York (NY), the United States of America (USA)). Spearman’s correlation coefficient was applied to determine associations between results from the traditional sucrose preference test and our iSPT and its further outcome measures. Mann-Whitney test was used for analyzing group differences for non-normally distributed variables (e.g. the sucrose preference score) between METH and naive monkeys. Student’s t-test was used for analyzing group differences for normally distributed variables (e.g. total liquid consumption) between METH and naive monkeys. Due to the limited sample size, Spearman’s correlation coefficient was applied to determine associations between the duration of abstinence and outcomes. Analysis of covariance (ANCOVA) test was used to analyze group differences for the new indicators between METH and naive monkeys, with age and duration of abstinence treated as covariates. The Mantel-Cox test was used to analyze group differences in latency data between the two groups. Two-sided p-values less than 0.05 were considered statistically significant.

## 3 Results

### 3.1 Correlations between outcome variables

We first compared behavior in sessions to validate our iSPT and investigate the relationships among the outcome variables by correlating sucrose preference scores obtained from the SPT with those measured using the iSPT in 5 METH exposed and 16 naive control monkeys (see here, 3.1.). The characteristics of monkeys in METH group are shown in **Table 1**. For direct group comparisons between METH exposed and naive control monkeys, we selected the 5 oldest monkeys from the pool of 16 naive monkeys for higher age to better match their ages to that of the METH group (see below, 3.2.). Age, sex and further characteristics of these 5 naive and 5 METH monkeys are presented in **Table 2**.

**Table 1:** Characteristics of the METH group.

Subject	First date of administrating	Last date of administrating	Drug dosage (mg/kg)	Duration of abstinence
110135	2021/07/08	2021/11/16	57.604	12 months
110127	2019/10/30	2019/12/16	25.336	44 months
110131	2019/10/25	2020/01/17	32.956	43 months
110579	2022/06/27	2023/02/14	115.548	6 months
1102035	2022/07/18	2023/05/19	115.712	3 months

**Table 2:** Characteristics of the 5 long-term abstinent monkeys previously exposed to methamphetamine (METH) and the 5 METH naïve monkeys (Mean ± SD).

Group	Age	Sex	Drug dosage (mg/kg)	Duration of abstinence (month)
METH	12.3 ± 0.3	Male	69.43 ± 43.83	21.60 ± 20.26
Naive	8.6 ± 3.3	Male	–	–

Firstly, we tested the validation of iSPT. As shown in **Supplementary Figure S1**, we observed a significant positive correlation between the sucrose preference scores (defined as the amount of sucrose solution divided by the total fluid intake per session) obtained from the SPT and those derived from the iSPT (r = 0.45, df = 61, p < 0.001).

Within the iSPT, significant correlations were found between the amount of sucrose solution intake as a percentage of overall fluid intake per session (i.e. the sucrose preference score) and sucrose-related preference (defined as the proportion of time spent on the sucrose-related bottle assessed) during the first 5 minutes (r = 0.85, df = 46, p < 0.001), 10 minutes (r = 0.88, df = 46, p < 0.001) and 1 hour of each test session (r = 0.90, df = 46, p < 0.001).

Furthermore, sucrose preference score was significantly correlated with the latency of the establishment of stable sucrose-preference (r = -0.76, df = 46, p < 0.001). On the other hand, sucrose preference score was neither significantly correlated with the water-sucrose switch latency (r = -0.30, df = 20, p = 0.18) nor with switch times (r = 0.04, df = 46, p = 0.79). Also, the latency of the establishment of stable sucrose-preference was neither significantly correlated with the number of switches per session (r = 0.15, df = 46, p = 0.30) nor the water-sucrose switch latency (r = 0.05, df = 20, p = 0.84). However, the water-sucrose switch latency was significantly correlated with the number of switches between bottles (r = -0.50, df = 20, p = 0.02).

### 3.2 Group comparisons between long-term abstinent METH exposed and naive monkeys

#### 3.2.1 The METH group showed lower sucrose preference scores than naive group

**Figure 2:**
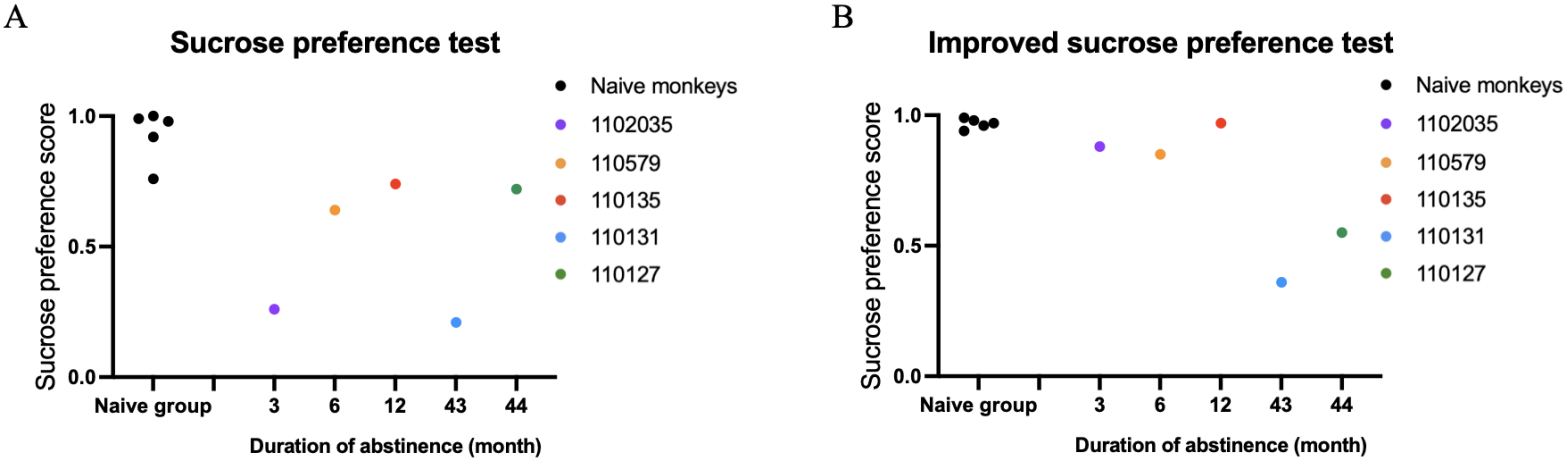
Results of the traditional sucrose preference test (SPT) and the improved sucrose preference test (iSPT) in all individuals of n=5 control and n=5 METH abstinent monkeys. **(A)** The sucrose preference score in the SPT. **(B)** The sucrose preference score in the iSPT.

We then conducted individual-level comparisons between 5 METH-exposed monkeys and 5 naïve control monkeys. The results of the traditional sucrose preference test (SPT) and the iSPT in all individuals of n=5 control and n=5 METH abstinent monkeys were shown in the **Figure 2**.

**Figure 3:**
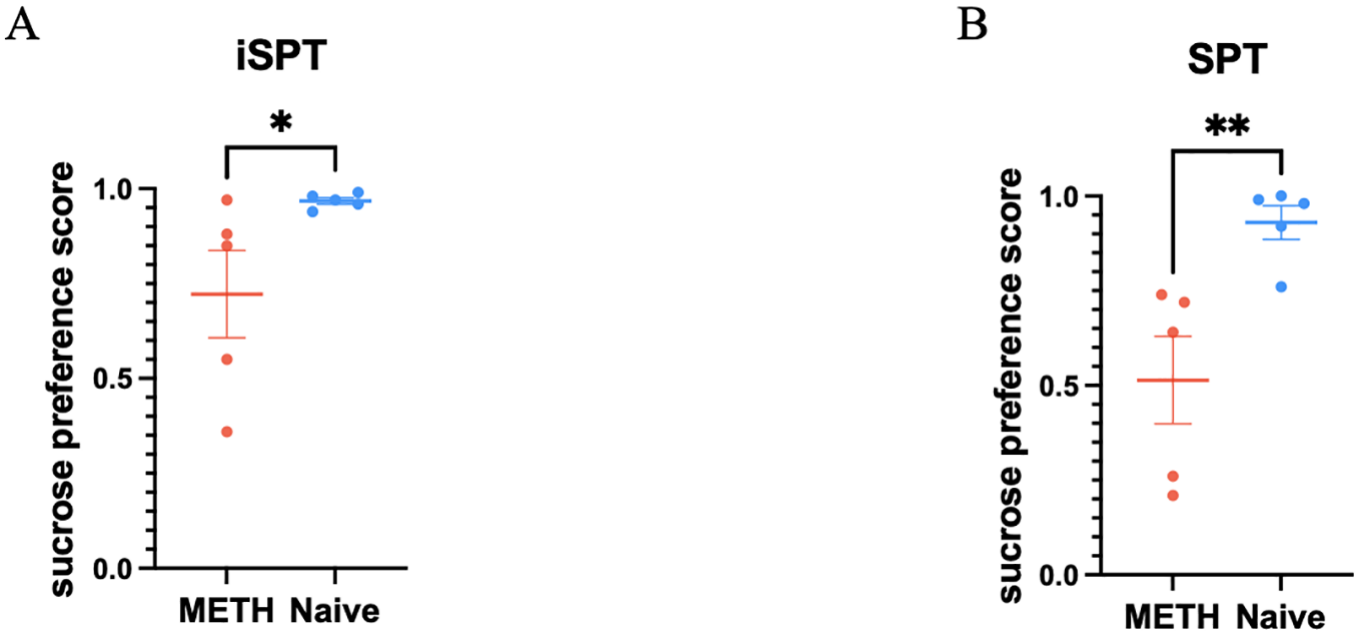
Comparisons of sucrose preference scores obtained from the improved sucrose preference test (iSPT) and the traditional sucrose preference test (SPT) between the two groups, comprising 5 METH abstinent monkeys and 5 age matched control monkeys. **(A)** Sucrose preference scores obtained from iSPT in individual monkey for both groups. *p < 0.05. Mean ± SEM. Mann-Whitney test. **(B)** Sucrose preference scores obtained from SPT in individual monkey for both groups. **p < 0.01. Mean ± SEM. Mann-Whitney test.

There was a significant group difference between the long-term abstinent monkeys who had been METH exposed and the drug naive monkeys when measuring sucrose preference scores with the iSPT (z = -2.10, p = 0.036, **Figure 3A**). We also found a significant difference between two groups with SPT (z = -2.61, p = 0.009, **Figure 3B**). On the other hand, total liquid consumption did not show a significant difference between two groups (t = -2.35, p = 0.193).

Then we analyzed data from video recording. Because of camera angles, we only got video data in three monkeys (110127, 110131, 1102035) in the METH group and four monkeys in the naïve group. We found that there was no significant group difference regarding water-sucrose switch latency, the latency to the first drink, switch times or the latency of the establishment of stable sucrose-preference for two groups in individual monkey; results remained non-significant when we covaried for age and duration of abstinence (see **Supplementary Figure S2** and **Supplementary Table S1**). There were also no significant differences in the latency to the first drink (p = 0.075), water-sucrose switch latency (p = 0.221) and the latency of the establishment of stable sucrose-preference (p = 0.084) between the two groups, as determined by Mantel-Cox analysis.

#### 3.2.2 The correlation between indicators in two groups with iSPT

Within these seven monkeys that had video recordings, as shown in **Supplementary Figure S3**, we found that water-sucrose switch latency was negatively correlated with sucrose preference scores (r = -0.82, df = 5, p = 0.023) and switch times (r = -0.86, df = 5, p = 0.014). There was also a significant correlation between the sucrose preference score and the latency of the establishment of stable sucrose-preference (r = -0.78, df = 5, p = 0.041).

#### 3.2.3 No significant correlation between the duration of abstinence and sucrose consumption

We found that the sucrose preference scores obtained from iSPT was not significantly correlated with the duration of abstinence (r = -0.60, df = 3, p = 0.285). The sucrose preference score in the traditional SPT was also not significantly correlated with the duration of abstinence either (r = 0.20, df = 3 p = 0.747).

## 4 Discussion

The key finding of this study was that monkeys exposed to methamphetamine (METH) injections displayed impaired consumption to natural rewards in spite of long-term abstinence from METH use. These findings supported the hypothesis of long-lasting impairments in consumption of natural rewards in individuals previously exposed to psychostimulants and potentially other drugs of abuse (8, 23–25). In turn, detoxified individuals might relapse to drug seeking and intake when natural rewards failed to attract approach and consumption, thus increasing the relapse risk and increasing the need for adequate therapeutic interventions (24–26).

With respect to natural reward consumption, the METH abstinent monkeys displayed reduced sucrose intake proportion both when assessed with a traditional SPT as well as the iSPT. The iSPT provides more time for monkeys to get used to the experimental conditions, suggesting that reduced reward consumption in METH exposed monkeys is not simply due to difficulties in adjusting to the test conditions. Sucrose consumption proportion assessed with our new task was significantly correlated with sucrose intake proportion measured with a traditional SPT as well as the time period to establish stable sucrose-preference. The latter is a surrogate marker for place preference, further validating our iSPT. Interestingly, we observed strong correlations between sucrose preference scores and sucrose-related place preferences during the first five or ten minutes of each test session and the overall consumption during this time period, suggesting that sucrose preference can be measured parsimoniously with our modified task. We also found that the sucrose preference score was not correlated with the duration of abstinence. This is noteworthy in light of prior work using self-report measures of anhedonia showing that anhedonia tended to diminish with abstinence from METH use (27).

Regarding new outcomes, the time period after which monkeys switched from first consuming water to sucrose was significantly correlated with the overall number of switches. Monkeys with longer latency to seek sucrose had fewer switch times. The sucrose preference score was negative correlated with the latency to establish sucrose-preference, which may both reflect how much a reward is “liked”. The water-sucrose switch latency may reflect how much a reward is “wanted”. Interestingly, the switch times was negative correlated with the water-sucrose switch latency. One study showed that rats given exposure to food rich in fat and sugar increased the latency to engage in licking and reduced alternations between spouts in the two-choice preference tests, which may indicate similar associations between these measures (28). We hypothesize that the switches in this experimental paradigm could be interpreted as a behavioral measure of motivation, suggesting that this paradigm has the potential to reflect deficits in motivation (29). However, additional evidence is needed to support the correlation between the new indicators and the potential underlying processes. For example, progressive ratio (PR) schedules are commonly utilized as a paradigm for assessing motivation, wherein the effort required to obtain a reward is incrementally increased (30–32).

Several limitations of this study need to be addressed. First, a cross-sectional design was employed, while a longitudinal study could be better to investigate the process of dynamic changes within the individuals during long-term abstinence. This would be specifically informative, as some clinical investigations demonstrated that individuals with psychostimulant use disorders displayed changes in the ability to experience pleasure during short- and long-term abstinence compared with control groups (33, 34). A study has demonstrated that the some of the structural changes induced by cocaine, which related to cognitive functions tested by stimulus reversal learning, remained despite long-term abstinence in monkeys (35). Furthermore, our investigation exclusively focused on male monkeys, thus precluding any comparisons or insights regarding potential sex-specific differences. The control group did not undergo the same surgical procedures or receive intravenous saline injections as the METH group, which could have impacted their response to natural rewards. Finally, we did not find a significant difference in the video-based variables between the two groups, which could be attributed to the limited sample size in these groups.

Altogether, we observed significantly reduced consumption of a non-drug reward (here sucrose) in monkeys with long-term abstinence from METH. Sucrose intake and the duration to establish a stable preference for sucrose consumption were significantly correlated, while significant association was observed between the overall number of switches between bottles and the latency to switch from consuming water to sucrose. With respect to translation, impaired consumption of non-drug rewards may increase the relapse risk in humans and require targeted interventions (24, 36).

## 5 Conclusion

These results show reductions in natural reward consumption during long-term METH abstinence.

## Data Availability

The original contributions presented in the study are included in the article/**Supplementary Material**. Further inquiries can be directed to the corresponding authors.
