# Supplementary material for: Decreased consumption of natural rewards in rhesus monkeys with prolonged methamphetamine abstinence
Source: Front Psychiatry. 2024 Sep 6;15:1446353. doi: 10.3389/fpsyt.2024.1446353 (PMC11412841; doi:10.3389/fpsyt.2024.1446353)
Supplement: Supplementary file 1 [file DataSheet1.pdf]

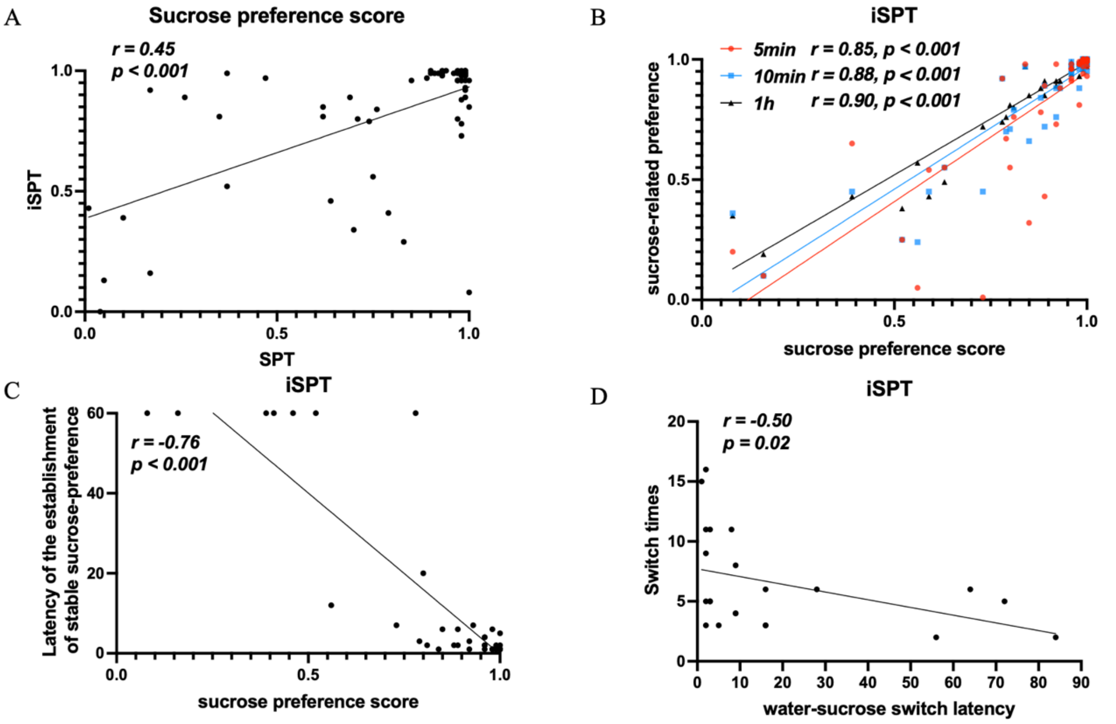

Figure S1

Results of the traditional sucrose preference score (SPT) and the improved sucrose preference test (iSPT) in all sessions of n=16 control and n=5 METH abstinent monkeys

A) *The correlation of SPT and iSPT results in all 16 control and 5 METH exposed monkeys, $r = 0.45$, $p < 0.001$ (Spearman's correlation coefficient).*

B) *The correlations between sucrose preference scores and the sucrose-related preference in shorter observation periods: 5 minutes ($r = 0.85$, $p < 0.001$), 10 minutes ($r = 0.88$, $p < 0.001$) and 1 hour ($r = 0.90$, $p < 0.001$) in all sessions of 16 (13 control and 3 METH) monkeys with video data. Spearman's correlation coefficient.*

C) *The correlation between the sucrose preference score and the latency of the establishment of stable sucrose-preference with iSPT in all sessions of 16 monkeys with video data, $r = -0.76$, $p < 0.001$. Spearman's correlation coefficient.*

D) *The correlation between water-sucrose switch latency and switch times with iSPT in all sessions of 16 monkeys with video data, $r = -0.50$, $p = 0.02$. Spearman's correlation coefficient.*

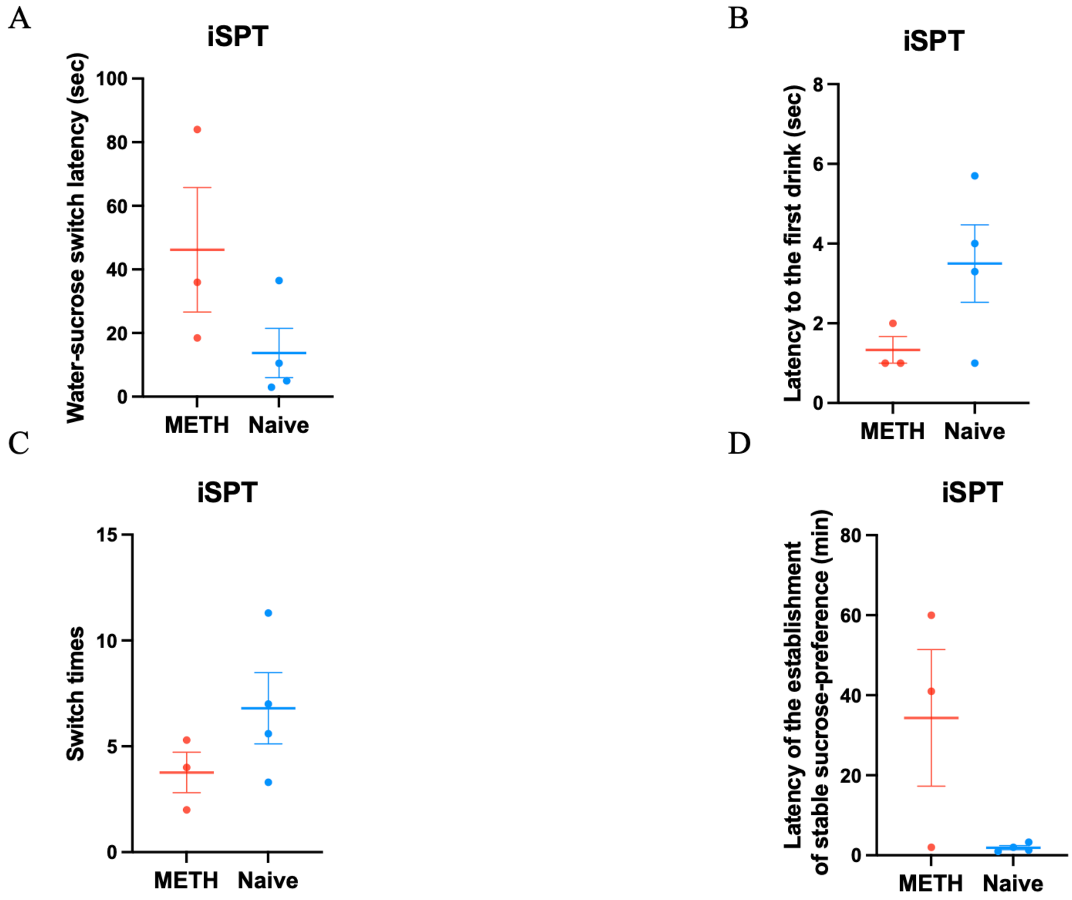

Figure S2

New outcomes obtained from the improved sucrose preference test (iSPT) between the two groups, comprising 4 control and 3 METH abstinent monkeys in individual data.

A) Water-sucrose switch latency obtained from iSPT in individual monkey for both groups. Mean ± SEM.

B) Latency to the first drink obtained from iSPT in individual monkey for both groups. Mean ± SEM.

C) Switch times obtained from iSPT in individual monkey for both groups. Mean ± SEM.

D) Latency of the establishment of stable sucrose-preference obtained from iSPT in individual monkey for both groups. Mean ± SEM.

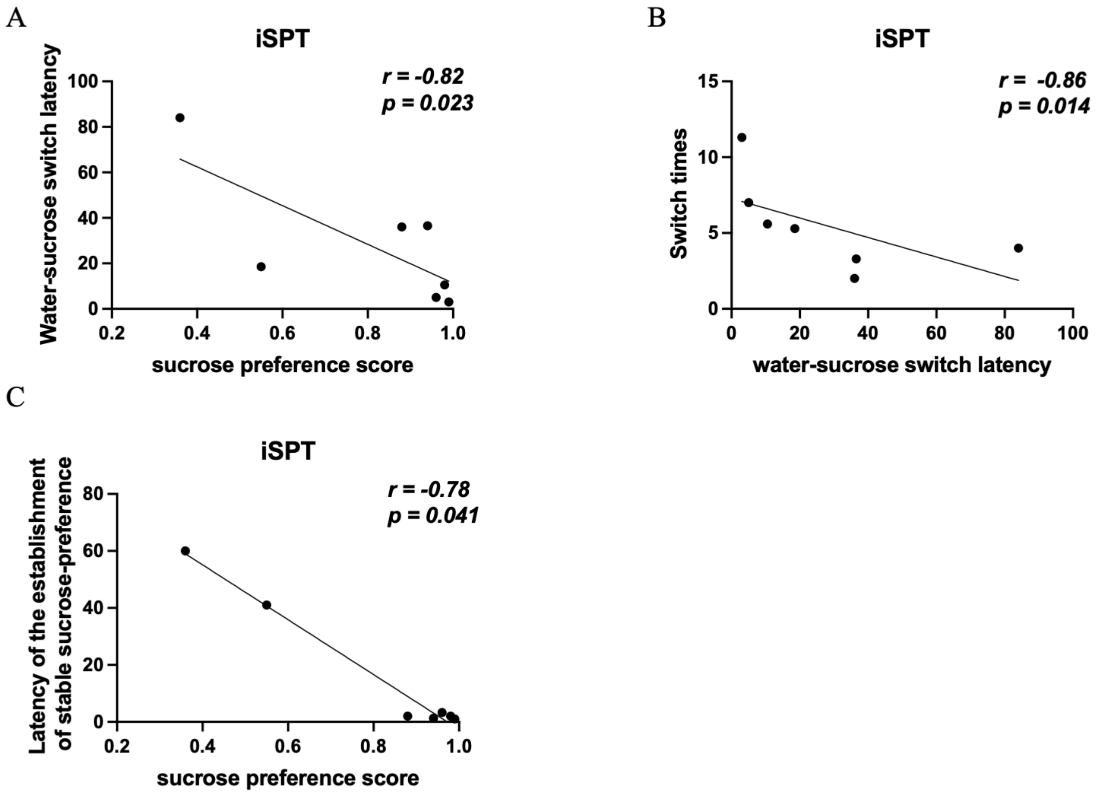

Figure S3

Results in videotaped individuals of the age-matched METH exposed (n=3) and control (n=4) monkeys

A) The correlation between the water-sucrose switch latency and the sucrose preference score with iSPT, r = -0.82, p = 0.023. Spearman's correlation coefficient.

B) The correlation between the water-sucrose switch latency and switch times with iSPT, r = -0.86, p = 0.014. Spearman's correlation coefficient.

C) The correlation between the latency of the establishment of stable sucrose-preference and the sucrose preference score with iSPT, r = -0.78, p = 0.041. Spearman's correlation coefficient.

Table S1

Results of new outcome obtained from the improved sucrose preference test (iSPT) of the naive versus METH abstinent group (Mean ± SD)

	Group	
	METH	Naive
Latency to the first drink (sec)	2.00±1.00	3.50±1.95
Water-sucrose switch latency (sec)	46.17±33.91	13.75±15.49
Switch times	3.78±1.67	6.80±3.37
Latency of the establishment of stable sucrose-preference (min)	34.33±29.57	1.90±1.02

**All group differences were all not significant, independent of whether they were analyzed using ANCOVA or the Mantel-Cox test.*
